# Unveiling Key Factors in Child Development: From Breastfeeding to Teachers’ Care in Brazilian Early Childcare Centers

**DOI:** 10.3390/ijerph22071158

**Published:** 2025-07-21

**Authors:** Alessandra Bombarda Müller, Helena Cristina V. S. Vieira, Carolina Panceri, Glauber Carvalho Nobre, Nadia Cristina Valentini

**Affiliations:** 1 Physiotherapy Department, School of Health, Universidade do Vale do Rio dos Sinos, Cristo Rei, Sao Leopoldo 93022-750, Rio Grande do Sul, Brazil; abombarda@unisinos.br; 2 Hospital de Clínicas de Porto Alegre, Ramiro Barcelos, Porto Alegre 90035-903, Rio Grande do Sul, Brazil; helena.cristina.vieira@hotmail.com (H.C.V.S.V.); cpanceri@hcpa.edu.br (C.P.); 3 Department of Physical Education and Sports, Federal Institute of Education, Science and Technology of Ceará, Fortaleza 60020-181, Ceará, Brazil; glauber_nobre@hotmail.com; 4 School of Physical Education, Physiotherapy and Dance, Universidade Federal do Rio Grande do Sul, Porto Alegre 90690-200, Rio Grande do Sul, Brazil

**Keywords:** child development, childcare center, cognitive development, language development, motor development

## Abstract

This study aimed to compare cognitive, language, and motor development outcomes among children attending public and private Early Childcare Centers (ECCs), considering birth factors and family and daycare environments. Additionally, it examined the proximal and distal factors influencing children’s development. Cognitive, language, and motor skills were assessed in the children, along with evaluations of ECC quality, teacher practices, and knowledge of child development. Results indicated that children enrolled in public ECCs achieved higher scores in cognitive and language development, despite coming from families with lower socioeconomic status and having lower birth weights. They also benefited from longer periods of breastfeeding. Teachers in public ECCs demonstrated greater daily practices, providing enhanced movement opportunities for children. Private ECCs offered more suitable outdoor spaces, whereas public ECCs had better indoor spaces. Regression analysis revealed that daily practice, teachers’ experience, and the availability of gross motor toys explained 41% of the variance in motor development. The duration of breastfeeding explained 24% of the variance in cognitive development. Teachers’ knowledge about children’s development and attendance at public ECCs explained 31% of the variance in language development. These findings underscore the importance of prioritizing teacher education in both public and private ECCs to optimize children’s overall development.

## 1. Introduction

The foundational years of a child’s life are critical for cognitive, language, and motor development, which are significantly influenced by risk factors at birth (e.g., birth weight, gestational weight, clinical adverse outcomes), as well as by the child’s interactions with their immediate environment and their caregivers. Given the rapid brain growth and maturation of nervous structures during this period, the quality of Early Childcare Centers (ECCs) plays a crucial role that extends far beyond meeting basic needs, such as hygiene, nutrition, and sleep [1]. It is imperative to foster an environment that encourages interaction with both adults and peers and provides opportunities for exploration under the guidance of trained professionals [1,2,3]; this approach is essential for nurturing development during these formative years.

Traditionally, the primary responsibility for nurturing early development has resided with the family, especially the mother. However, with the increasing need for dual-income households, caregiving responsibilities are frequently outsourced to ECCs [4], often requiring a premature end to exclusive breastfeeding, an essential protective factor of development, and altering the traditional caregiving dynamic [4,5]. Consequently, infants are entrusted to caregivers in ECCs at a younger age [5]. This shift in childcare underlines the growing reliance of parents on ECCs. How these changes affect children’s development is still uncertain, especially in low- and middle-income countries (LMICs), where there is a pronounced gap in evidence on child opportunities and development outcomes within these settings [6].

ECCs are uniquely positioned to mitigate the developmental risks associated with socioeconomic disadvantages [7,8]. Lower socioeconomic status is often associated with reduced parental formal education levels, which can adversely affect a child’s developmental trajectory [9]. ECCs not only provide crucial care but also serve as a vital strategy to enhance the quality of life for children from economically challenged backgrounds by compensating for the lack of resources and educational opportunities within the home [10]. Delivering high-quality care in ECCs, characterized by safe learning interactions within a secure environment handled by skilled and supportive staff, is fundamental [2,3,11]. The adequacy of facilities [8], optimal caregiver-to-child ratios [12], structured routines [13], access to educational toys [9,14], and staff awareness of developmental milestones [3] are key indicators of ECC quality. These factors collectively support children’s development, supplementing familial educational efforts [2,10].

Despite the recognized importance of ECCs, disparities exist between the resources and quality of care provided in public versus private centers, particularly in LMICs like Brazil. In Brazil, public ECCs are often the responsibility of the government and usually have limited coverage, equipment, professional training, and resources [14,15], whereas private ECCs frequented by middle- and high-income families have far greater human and material resources [16]. These disparities can significantly impact developmental outcomes for children [17]. However, there remains a significant knowledge gap regarding how these differences in ECC quality and resources translate into developmental outcomes, especially in LMICs such as Brazil. While previous studies have demonstrated that high-quality ECCs can mitigate the negative effects of socioeconomic disadvantages on child development, few have directly compared public and private ECC settings within these contexts. It is still unclear to what extent disparities in resources and caregiving practices translate into differences in cognitive, language, and motor development, particularly when considering the combined influence of birth risks, family context, and daycare environment factors. Addressing this gap is crucial to inform policies and interventions that aim to reduce developmental inequalities in early childhood.

This study aimed to bridge this knowledge gap by first comparing cognitive, language, and motor development outcomes among children in public and private ECCs, considering birth risk factors, family context (e.g., socioeconomic status, maternal education, breastfeeding duration), and daycare environment (e.g., staff experience, daily practices, and knowledge, and developmental opportunities). Additionally, it examined the proximal and distal risks and protective factors affecting children’s development. It was hypothesized that children in private ECCs will exhibit higher developmental outcome scores, influenced by better-informed childcare practices and richer developmental opportunities.

## 2. Materials and Methods

### 2.1. Participants

This study involved children, teachers, and assistants from both public and private Early Childcare Centers (ECCs) in southern Brazil using a multistage cluster design. The study adhered to ethical research principles outlined in Resolution 466/12 of the National Health Council. The research was approved by the university’s ethical committee of the Federal University of Rio Grande do Sul (CAAE: 35035014.6.0000.5347; Number 850.949/2018-10-29). Individual informed consent was obtained from children’s parents and all professionals enrolled in the study; all ten ECCs participating in the study also provided their institutional informed consent.

In the first phase of selection, ten ECCs were chosen at random from a list of approximately 100 centers, both private and public, accredited by the local education board to serve full-time children aged zero to twenty-four months from low- and mid–low-income families. The principal researcher obtained consent for participation by reaching out to the education board and the administrators of these institutions, all of whom agreed to participate.

In the second phase of selection, children aged 6 to 18 months who attended the ECCs daily and full-time (eight hours a day, five days a week, for at least six months) were randomly selected from each classroom among those meeting the inclusion criteria. These inclusion criteria aimed to ensure that the opportunities provided for the children’s development were mainly occurring within the collective educational setting. Random selection was used to ensure a representative sample within each ECC while maintaining feasibility in terms of assessments and data management. Including all eligible children would have exceeded the logistical and financial capacities of the study. Moreover, random sampling minimized potential selection bias and allowed findings to be generalizable within similar ECC contexts. A total of 90 children met these criteria and were selected for the study; children with disabilities were excluded. Teachers and assistants became part of the study only if the children in their care were selected. To ensure clarity and understanding of the study’s aims and procedures, the principal researcher conducted meetings with professionals and parents at the ECCs.

The sample size was determined using the G*Power program (version 3.1). For primary analysis comparing two groups using one-way ANOVA, an effect size of 0.30 (moderate effect), power level of 0.80, and a significance level of *p* = 0.050 indicated that a minimum of 90 children was required. Additionally, for the planned hierarchical multiple linear regression analysis with fifteen predictors (controlling for age), the required sample size to detect a moderate effect (Cohen’s ƒ^2^ = 0.30) with a power (1 − β) ψ = 0.80 at a significant level of *p* = 0.050 was 77 children. Therefore, the study aimed to recruit at least 90 children to meet the more conservative sample size estimate for the ANOVA analysis while also ensuring adequacy for regression models.

Out of the initially selected 90 children, 3 were diagnosed during the study with a genetic syndrome, neurological impairment, and visual and auditory deficits and were therefore excluded. Additionally, three other children did not attend the ECCs on the designated assessment days, and after three unsuccessful attempts to reach their parents, these children were also excluded. Consequently, the final sample consisted of 84 children.

To ensure the adequacy of this final sample despite these exclusions, a post hoc analysis was conducted. This analysis confirmed that the achieved sample of 84 children was sufficient to achieve the power (0.80) to detect a moderate effect size (Cohen’s ƒ^2^ = 0.31) at a significance level of *p* ≤ 0.050 in the regression analysis and remained near the originally estimated sample size needed for ANOVA group comparisons. Thus, the final sample was adequate to address the study’s aims while maintaining statistical validity.

Therefore, the sample consisted of 84 children, with 41 boys and 43 girls, evenly distributed across public and private groups. Children’s ethnicities, as per the Brazilian governmental categorization [18], were also similar across public and private ECCs. Just over half of the children were delivered via cesarean section (overall sample 51.2%), with no reported complications during delivery. Children attending public and private ECCs came from families of different socioeconomic status—SES. Most children from public ECCs were from families with low SES, while most children from private centers were from middle–low SES [19]. None of the children lived below the Brazilian poverty line [18].

Ten teachers and fourteen assistants who were responsible for the children’s care took part in the study. Each teacher was paired with a teaching assistant, with six teachers having full-time assistants (n = 6) and four teachers having part-time assistants (n = 8). All teachers held teaching degrees and had undergone specific training to care for children in this age group. Similarly, all assistants had completed at least a high school education and received training for childcare. Both teachers and assistants were exclusively female, aged between 25 and 35, and had several years of experience working with children.

Each ECC had multidisciplinary administrative teams responsible for implementing the pedagogical plan and providing support to educators. The ratio of one teacher to one assistant for every 8 to 12 children adhered to the recommendations of the city board of education. This ratio was determined based on considerations of the pedagogical plan and the physical space available in the ECCs, ensuring that there were no more than six children per adult and a maximum of eighteen children per teacher for children aged 0 to 2 years. The ECCs’ classrooms were organized based on the children’s ages, with separate areas designated for infants aged 6 to 12 months and toddlers aged 12 to 18 months.

### 2.2. Instruments

#### 2.2.1. Socioeconomic Status

The Brazilian Economic Classification Criteria [19] questionnaire was used to assess family SES. This tool evaluates SES by considering factors such as the number of automobiles and rooms in the household, ownership of appliances (e.g., freezer, washing machines, microwave, computers), employment of housekeepers, the education level of the household head, access to public services (e.g., piped water, paved streets), and monthly income. Based on the responses, families received a score ranging from 0 to 100 points, which, along with the family’s monthly income, determined their classification into one of six categories: D/E (0–16 points, income up to BRL 826.41), C2 (17–22 points, income up to BRL 1894.95), C1 (23–28 points, income up to BRL 3194.33), B2 (29–37 points, income up to BRL 5721.72), B1 (38–44 points, income up to BRL 10,788.56), and A (45–100 points, income over BRL 22,749.24).

#### 2.2.2. Sociodemographic Survey

Parents were provided with a survey to fill out, which collected information on the infant’s age, sex, ethnicity, gestational age at birth, delivery method, and any complications occurring before, during, or after birth. It also inquired about the infant’s APGAR score, general health and care practices, and the duration of breastfeeding.

#### 2.2.3. Children Development

The Bayley Scales of Infant and Toddler Development—Third Edition (BSITD-III) [20] was used to assess children’s motor, cognitive, and language development. This tool assesses infants’ performance relative to their age, adjusting for corrected age in the case of premature birth up to 24 months. Widely acknowledged as the gold standard for assessing child development, the BSITD-III provides composite scores and the categorization of performance. Scores of 85 or higher fall within the typical development range, while delays are categorized as severe (composite scores below 55), moderate (composite scores from 55 to 69), and mild (composite scores from 70 to 84) [21].

#### 2.2.4. Teachers’ and Assistants’ Knowledge

The Knowledge of Infant Development Inventory (KIDI) [22], adapted for Brazilian children [23], was used to assess caregivers’ understanding of child milestones. The KIDI comprises 75 questions organized into four behavioral categories: care (i.e., beliefs, behaviors, and responsibilities), developmental milestones (i.e., motor, perceptual, and cognitive skills), principles (i.e., typical and atypical development), and health (i.e., nutrition and prevention of accidents). The total score, ranging from 0 to 100, is calculated by the ratio of correct answers to the total number of questions for each age group.

#### 2.2.5. Teachers’ Practice

The Daily Activities of Infant Scale (DAIS) [24] was used to assess the teachers’ practice. The DAIS comprises eight dimensions (i.e., feeding, bathing, changing clothes, nap time, quiet and active games, walking, and sleep). It uses pictures to identify the postures most assumed by the infant during the day, facilitated by the caretaker. Positions requiring less anti-gravitation control receive lower scores (A: low stimulation = 1; B: average stimulation = 2), while those demanding greater challenges receive higher scores (C: more stimulation = 3). Raw scores are provided for each dimension, and total scores range from 10 to 24. Only the teachers completed the scale, considering one for each child. In this study, the DAIS was also used to evaluate children aged 12 to 18 months. Adaptation of questions was deemed unnecessary, with expert consensus indicating that the questions were appropriate for this age group, reaching 97% agreement. The DAIS demonstrated satisfactory internal consistency indices (α values ranging from 0.80 to 0.85), and test–retest reliability results showed strong Intraclass Correlation Coefficients (ICC values > 0.90) for assessing the daily care of children aged 12 to 18 months.

#### 2.2.6. Childcare Centers’ Opportunities for Development

The Affordances in the Daycare Environment for Motor Development (ADEMD) [25], adapted from the AHEMD [26] and validated for Brazilian children [27], was used to assess the opportunities available for motor development at the ECCs. The ADEMD assesses the potential for children’s development by examining the physical space (e.g., texture, inclinations, stairs, furniture, floor), the variety of daily activities (e.g., postures, use of devices, games), and the availability of fine and gross motor toys. The leading researcher collected the ADEMD information.

### 2.3. Procedures

The university’s research committee approved the study, which received further endorsement from the education secretary, who provided a list of institutions that enrolled children full-time. The leading researcher carried out the random process to select ten ECCs. Each selected ECC agreed to participate in the research. The researcher also conducted a random selection of children within these institutions. To engage the parents, the researchers organized meetings to outline the study’s aims and the informed consent process. Consent forms were distributed to be taken home. For the parents who consented, the researchers detailed the protocols and questionnaires involved in the study.

Sociodemographic questionnaires were given to parents to complete at home and return to the researcher. All teachers and assistants at the ECCs consented to participate, signing the informed consent forms. They were then briefed on completing the KIDI [22] and the DAIS [24] individually. The principal researcher collected data for the ADEMD [25] at each ECC on the initial visit. The data collection took approximately six months and was conducted in the first semester with the support of two doctoral students and one undergraduate student. All surveys were completed prior to assessing child development.

The BSITD-III [21] assessments were carried out in quiet rooms within the ECCs, under the supervision of teachers. All assessments were administered by a licensed physical therapist with over 20 years of clinical experience, more than 5 years of experience using the BSITD-III, and formal training in its administration and scoring. Each assessment lasted about 60 min per infant. These sessions were recorded to enable later scoring by an independent examiner, who was also a licensed physical therapist with 10 years of clinical experience and verified training in the use of the BSITD-III. This ensured that the process included an inter-rater reliability analysis using the Intraclass Correlation Coefficient (ICC) for motor, cognitive, and language development scores. The ICC results showed high inter-rater reliability for motor (ICC = 0.98, 95% CI = 0.95–0.99), cognitive (ICC = 0.98, 95% CI = 0.97–0.99), and language (ICC = 0.95, 95% CI = 0.93–0.97) scores [28].

### 2.4. Data Analysis

Mean, standard deviations, and correlations are presented. Chi-square tests and one-way ANOVA were used to compare public and private ECCs. Cohen’s *f (f)* was used as the ANOVA effect size index with recognized cut-offs (Cohen’s *f* effect size: *f* small = 0.10 to 0.24; *f* moderate = 0.25 to 0.39; *f* large ≥ 0.40) [29].

Pearson and Spearman (when appropriate) correlation coefficient tests were utilized adopting recognized cut-offs (very weak *r* < 0.10; weak r = 0.10 to 0.30; moderate *r* = 0.30 to 0.50; moderate to strong *r* = 0.50 to 0.70; strong *r* = 0.70 to 0.90; very strong *r* > 0.90) [28]. Hierarchical multiple regression was conducted to test if the independent variables significantly explained the motor, cognitive, and language outcomes. Variables groups were nested in three models. The standard theoretical procedure for cumulative hierarchical regression was applied, whereby variables from earlier models were retained in all subsequent steps to assess the incremental contributions of newly added predictors [28]. Initially, the first model included age, birth weight, birth height, gestational age, mechanical ventilation, APGAR 5° min, days in the neonatal intensive care unit, and length of breastfeeding as the predictors. In the second model, besides the first mentioned variables, teachers’ daily practice, teachers’ and assistants’ knowledge, teachers’ and assistants’ experience, and mothers’ formal education (high school and college) were also predictors. The last model included indoor and outdoor ECCs’ physical space, fine and gross motor toys, families’ monthly income, and ECC category (public and private).

The parameters were estimated using the least square method. The Kolmogorov–Smirnov test was used to examine the normality of residuals, and a Q–Q plot with standardized residuals from the models was used to assess normality visually. Homoscedasticity was tested using the Breusch–Pagan test and by examining the scatterplot of residuals. The independence of error distribution was examined using Durbin–Watson statistics; values between 1.5 and 2.5 indicated no linear autocorrelation in the data. Collinearity was controlled using the VIF (variance inflation factor) test; values above five were indicators of collinearity, and those variables were removed from the models. Akaike Information Criteria (AIC) and Bayesian Information Criteria (BIC) were used to compare the quality of models; adjusted R^2^ and ΔR^2^ were presented. Cohen’s effect size for hierarchical multiple regression (*f*^2^) was estimated adopting recognized cut-offs (*f*^2^ small = 0.02 to 0.15; *f*^2^ moderate = 0.15 to 0.34; *f*^2^ large ≥ 0.35) [29], and power values ≥ 0.80 were strong; α-level = 0.050 and two-tailed test were adopted. Three statistical software, SPSS (version 29), R studio (version 4.5.1), and Jamovi 2.4.5, were used.

## 3. Results

### 3.1. Public and Private ECC Comparisons

Table 1 presents the results for individual and contextual factors for children at public and private ECCs. The one-way ANOVA showed that children from public and private ECCs had similar results in several individual characteristics. However, birth weight and family monthly income were significantly higher for children from private ECCs. In contrast, breastfeeding was maintained longer for children in public ECCs, with moderate effect sizes. Additionally, teachers from public ECCs exposed children to more challenging postures during daily practices. Private ECCs had more suitable outdoor spaces, whereas public ECCs had more suitable indoor spaces.

Table 2 presents the group comparisons for children’s developmental outcomes. The results showed that children from public ECCs had significantly higher cognitive and language composite scores, with a small effect size.

**Figure 1:**
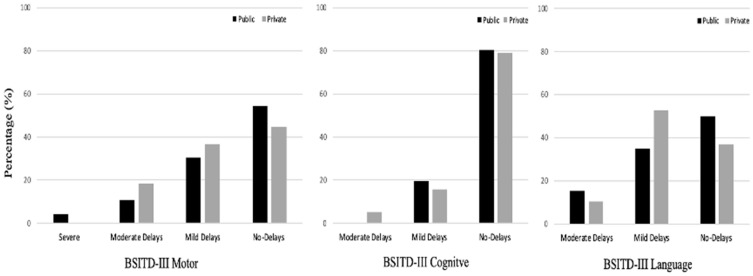
Prevalence of typical development and delay across public and private ECCs.

Regarding delay prevalence, although less prevalence of delays was observed for children in public ECCs in the motor and language categorizations of performance, no significant differences were found (*p* > 0.050). Approximately 50% of the children showed motor delays, 20% cognitive delays, and 55% language delays. Figure 1 presents the prevalence of delays (severe, moderate, and mild) and typical development [20].

### 3.2. Association of Risk and Protective Factors and Children’s Development

Several procedures were conducted to test the assumptions for the hierarchical regression analysis. The results showed that the normality of residuals was confirmed by the Kolmogorov–Smirnov (*p*-values > 0.05) test for all models; the Q–Q plot also visually confirmed the normality. The Breusch–Pagan test (*p*-values > 0.050) and a scatterplot of residuals indicated the homoscedasticity of the models. The Durbin–Watson statistics indicated the independence of error distribution (values between 1.5 and 2.1). The VIF test indicated collinearity (values from 6.1 to 21.5) for several variables (birth height, birth weight, mode of delivery, mechanical ventilation, APGAR 5^0^ min, days in the neonatal intensive care unit, teachers’ experience, and assistants’ experience). When age, birth height, APGAR 5^0^ min, days in the neonatal intensive care unit, and assistants’ experience variables were included, all the models reached non-collinearity. The hierarchical theoretical model was created using the assumptions established by Victora et al. (2015) [30], organizing all factors according to the proximity to the presented outcomes measured. We used a cumulative hierarchical regression approach, where variables from earlier models were kept in all subsequent steps to evaluate the added contribution of each new set of predictors [28]. The selection of the allocation levels of variables for the hierarchical model followed the logic of individual factors, factors in the context of child development, and factors of daily care of the child, enrolling individuals and experiences within this daily routine.

**Figure 2:**
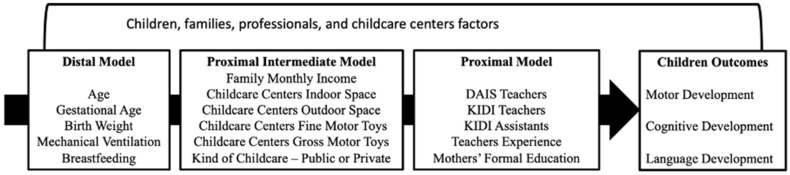
Hierarchical theoretical and analytical final model of factors associated with children’s development.

Three levels of variables were proposed and grouped in hierarchical blocks; those variables met all the statistical assumptions and therefore remained in the model. Figure 2 presents the model.

Table 3 presents the correlation matrix between all variables that remained in the model. Positive, negative, and moderate/strong to very weak correlations (*r*-values varying from −0.01 to 0.87) were observed among variables.

Regarding motor outcome, all three models were significant (model 1: F(5,70) = 2.26, *p* < 0.045; model 2: F(10,65) = 5.65, *p* < 0.001; model 3: F(16,59) = 4.30, *p* < 0.001). Breastfeeding significantly explained the variance in model 1 (β = 0.28, *p* = 0.017). Breastfeeding (β = 0.27, *p* = 0.008), mechanical ventilation (β = 0.23, *p* < 0.025), and teachers’ experience (β = 0.58, *p* < 0.001) explained motor variance in model 2. However, when adjusted for all other variables (model 3), teachers’ daily practices (β = 0.23, *p* < 0.046), teachers’ experience (β = 0.47, *p* < 0.001), and gross motor toys (β = 0.41, *p* = 0.012) better explained the variance in motor scores.

Concerning cognitive outcome, all three models were significant (model 1: F(5,70) = 4.47, *p* = 0.001; model 2: F(10,65) = 3.56, *p* < 0.001; model 3: F(16,59) = 2.53, *p* = 0.005). In model 1, breastfeeding (β = 0.41, *p* = 0.002) explained the variance. In model 2, breastfeeding (β = 0.38, *p* = 0.001), mechanical ventilation (β = 0.25, *p* < 0.025), and teachers’ experience (β = 0.23, *p* < 0.049) explained the variance. However, when adjusted for all other variables (model 3), only breastfeeding (β = 0.41, *p* = 0.002) better explained the variance in cognitive scores.

Concerning language outcome, all three models were significant (model 1: F(5,70) = 3.40, *p* = 0.008; model 2: F(10,65) = 3.67, *p* < 0.001; model 3: F(16,59) = 3.10, *p* < 0.001). Breastfeeding significantly explained the variance in model 1 (β = 0.38, *p* = 0.001). Breastfeeding (β = 0.30, *p* = 0.008) and teachers’ experience (β = 0.29, *p* = 0.014) significantly explained the variance in model 2. However, after adjusting for all other variables (model 3), teachers’ knowledge (β = 0.31, *p* = 0.030) and public ECCs (β = 0.75, *p* = 0.014) better explained the variance in language scores. The results of linear regressions are presented in Table 4.

## 4. Discussion

This study aimed to compare cognitive, language, and motor development outcomes between children in public and private ECCs, considering birth risks, family context, and daycare environment, and examined the risk and protective factors affecting development. It was hypothesized that children in private ECCs will exhibit higher developmental scores, which would be influenced by better-informed childcare practices and richer developmental opportunities.

### 4.1. Public and Private ECC(s) Comparisons: Children’s Birth Weight and Breastfeeding

Significant differences were observed between children in public and private ECCs regarding birth weight and breastfeeding duration. Children in public ECCs had significantly lower birth weights, with a moderate effect size noted. All preterm infants (n = 7) in public ECCs had low birth weights (below 2500 g), in contrast to just one out of five preterm infants in private ECCs, indicating increased risk factors for children in public settings. Low birth weight, often linked with prematurity, tends to be more common among children from lower socioeconomic backgrounds [9,31].

Interestingly, children attending public ECCs were breastfed for longer periods, about seven months, accomplishing WHO recommendations [32]—infants should be exclusively breastfed for the first six months of life, and breastfeeding should then continue alongside appropriate complementary foods up to two years of age or beyond, while those in private ECCs were breastfed for approximately five months. This contradicts prior research in Brazil indicating that children from disadvantaged families are less likely to be breastfed [33,34]. The observed trend in this study suggests a complex interplay of cultural, economic, and policy-driven factors. For economically disadvantaged families, exclusive and prolonged breastfeeding becomes a feasible, cost-effective alternative to expensive formula milk. Across Brazil, there exists a strong consensus on the importance of extended breastfeeding for a child’s health and development, deeply embedding it as a cultural norm [35]. This practice is further reinforced by Brazilian policies aimed, through public health campaigns, at promoting breastfeeding and the dissemination of its benefits. Moreover, the country’s maternity leave policy, offering 120 days of full pay, provides mothers with the opportunity for exclusive breastfeeding [36,37]. These factors combined underline the multifaceted influences on breastfeeding practices within the nation.

Another important factor to note is that, regardless of ECC type, over half of the sample was delivered by cesarean section. Over the past four decades, Brazil has experienced a steady and significant rise in cesarean section rates, with an increase from 38% to 48.4%. Although many women initially express a preference for vaginal birth, a large proportion ultimately have cesarean deliveries—often influenced by beliefs about reduced pain and the convenience of scheduling for medical staff, as many procedures occur during weekday daytime hours [38]. This pattern suggests that systemic and cultural factors, rather than maternal preference alone, contribute to Brazil’s high cesarean rates. To address this issue, health education initiatives and public policies are needed to raise awareness about the risks and benefits of different delivery modes and to promote informed, evidence-based decision-making among women and healthcare providers.

### 4.2. Public and Private ECC Comparisons: Families’ Monthly Income and SES

More children at public ECCs came from families with lower monthly incomes than those at private ECCs, although there were no significant differences in the mothers’ formal education levels across both groups. In Brazil, economically disadvantaged families often choose public childcare, if available, primarily due to financial constraints and the pursuit of social and educational benefits for their children. Public ECCs are known to offer essential services at no cost, including structured educational activities, socialization opportunities, nutritious meals, and, in some instances, routine health screenings [7,11,39,40]. Moreover, the public ECCs also provide a safe and supervised environment, reducing the risk of leaving children unattended at home or receiving inadequate care from older siblings. These offerings are particularly critical for low-income families that might otherwise find these services unaffordable.

However, despite Brazil’s progressive early childhood education policies [40,41,42], access to public ECCs for low-income families remains a significant challenge. The availability of public childcare has increased in recent decades, yet only 34% of those in need receive services regularly. Priority is given to children from families living in poverty, single-parent households, and those with mothers actively seeking or participating in the workforce [40]. This approach aims to support the most vulnerable segments of society, as demonstrated by the families in the current study.

The limited availability of public ECCs has compelled a small yet noteworthy fraction of low-income families (7.9%) to stretch their finances to enroll their children in private centers, potentially impacting their economic stability. This scenario highlights the critical role that public ECCs play in not only fostering child development and providing a safe and nurturing environment for children but also in enabling parents, particularly mothers, to pursue employment or further education. By addressing the childcare needs of low-income families, Brazil’s public childcare system could serve as a crucial mechanism in reducing social inequalities, underlining the importance of expanding access to quality childcare for all families.

### 4.3. Public and Private ECC Comparisons: Teacher’s Daily Practices

In the present study, teachers in the public ECCs provided better opportunities for children to experience challenging anti-gravitational postures during the daily care of the infant, during feeding, bathing, dressing, carrying, playing, and napping time routines, than teachers from private daycares. In public ECCs, teachers’ practices are shifting away from traditional routines that focus only on hygiene and feeding, which previously limited opportunities for interactions to nurture children’s development [9,11,14,24].

Public ECCs face resource constraints that require innovative, creative, and resourceful approaches to daily care by teachers. These educators engage children in activities that do not rely on costly equipment, instead using the available environment. They frequently arrange children in various floor positions for play, minimizing or avoiding traditional equipment such as baby comforts, highchairs, swing chairs, and fences. Simple items like pillows are utilized to support children in diverse postures, fostering engagement and exploration without the need for elaborate equipment. This innovative use of limited resources, coupled with enriched educational experiences for children from diverse socioeconomic backgrounds, underscores the adaptability and commitment of teachers in public ECCs to create an inclusive and stimulating learning environment.

### 4.4. Public and Private ECC Comparisons: Childcare Indoor and Outdoor Spaces

Public ECCs exhibited higher scores for attributes of indoor physical space, such as floor texture and equipment that facilitates children’s stand-up activities, including tables, chairs, benches, and stairs. In contrast, private ECCs demonstrated superior outdoor features, encompassing grass, dirt, sand, sloped surfaces, stairs, and slides. The quality of both indoor classrooms and outdoor areas is key in executing educational programs that bolster children’s development [4]. Access to appropriate physical environments is essential for promoting learning opportunities, ensuring mobility, and enhancing social interactions in ECCs.

The quality of childcare facilities is particularly vital for children from vulnerable families, who might lack adequate physical and developmental opportunities at home [2,17]. This study found that the environments where children spend significant time—eight hours per day, five days a week—were predominantly rated as good in both public and private ECCs. While public ECCs provided a broader range of indoor opportunities and private ECCs offered better outdoor facilities, both types of settings adhered to appropriate standards, ensuring children’s safety and supporting teacher functionality.

### 4.5. Public and Private ECC Comparisons: Children’s Development

Children in public ECCs exhibited higher cognitive and language scores than those from private centers, a finding that contrasts with prior research [14]. This discrepancy raises several considerations. One potential explanation is the implementation of more demanding daily routines in public ECCs, as indicated by the DAIS assessment. Such routines likely enhance children’s interactions in the activities on the floor, positively influencing cognitive and language development. Moreover, with public ECCs mostly serving children from socioeconomically disadvantaged backgrounds, teachers’ efforts may be particularly geared toward mitigating environmental factors that could impede cognitive and language development at home. These findings underline the importance of scrutinizing the childcare curriculum and guidelines within ECC programs for a comprehensive understanding of their impact.

The high incidence of developmental delays among infants in both public and private ECCs is concerning, with about 50% exhibiting motor and language delays and around 20% showing cognitive delays. These results align with previous Brazilian studies reporting developmental delay prevalences ranging from 20% to 50% [3,43]. Literature also suggests that the high number of children per room in ECCs poses a risk for development [14,15,16]. In this study, both public and private ECCs maintained a standard of one teacher and one assistant per room, with group sizes varying from small ratios (1 teacher to 6 children) to more crowded conditions (up to 1 teacher to 25 children). Such inadequate teacher–child ratios likely limit the necessary individual attention and interactions crucial for optimal development. Enhancing the quality of child–teacher interactions and fostering positive developmental outcomes may require a reduction in teacher–child ratios, a strategy that could beneficially affect care quality in both public and private ECCs in Brazil, thus helping to prevent developmental delays.

### 4.6. Risk and Protective Factors Associated with Children’s Motor Development: Teachers’ Daily Practices and Professional Experiences and Gross Motor Toys Available for Children

The hierarchical regression analysis concerning motor development outcomes revealed significant results across all three models. Initially, in the first two models, the variance in motor outcomes was explained by the length of breastfeeding, the need for mechanical ventilation, and teachers’ experience. However, when adjusting for the other variables, the factors that consistently remained significant in the final model were teachers’ daily practice (evaluated using the DAIS assessment), teachers’ years of experience, and the availability of gross motor toys for children. These variables collectively accounted for 41% of the variance in motor development outcomes.

Teachers’ daily practices significantly and positively explained children’s motor development. The results showed that the teachers who were more effective in providing children with opportunities to play actively, on the floor and standing alone when playing, challenged them to acquire demanding postures during feeding (sitting alone or in chairs with minimal support) and when changing clothes (standing up when dressing them), promoting higher motor development levels. The results clearly indicated that teachers with higher scores in their daily practices were the ones who rarely had the children under their care seated in baby comforts or restricted movement chairs, in the cribs, or even carrying them around. Children under their care spent most of the day on the floor, moving around, exploring, and climbing (upstairs, over objects, or up onto the furniture). The results are aligned with previous studies showing that teachers with high-quality daily practices encouraged child experience autonomy, positively impacting motor development [3,8,11].

Additionally, teachers’ experience also influenced children’s motor development. Experienced teachers promoted children’s motor skills through various activities, such as encouraging movement around the room, using furniture and equipment for standing and walking exercises, participating in obstacle courses, dancing, climbing stairs, and engaging in manipulative activities like fitting cubes into boxes and using small objects. Implementing these activities effectively for young children (6 to 18 months) in group settings requires strong leadership, classroom management skills, problem-solving abilities, and decision-making skills. Experienced teachers also demonstrated patience, clear communication, and attentiveness to individual needs while planning and delivering age-appropriate lessons or activities. These skills are honed over years of teaching experience, and experienced teachers might be more adept at ensuring the quality of daily childcare processes, compared to their less-experienced counterparts, potentially leading to higher developmental outcomes for children.

Consistent with prior research, the availability of gross motor toys was linked to enhanced motor development in children [7,9,44,45]. These toys, which necessitate the use of larger muscle groups, propel infants towards active engagement in physical activities such as crawling, walking, climbing, and reaching. This active engagement is crucial for strengthening muscles and developing coordination. The presence of toys within a child’s reach in their environment fosters opportunities for exploration and engagement in challenging postures, contributing to the creation of meaningful experiences [7]. Furthermore, gross motor toys facilitate environmental exploration and spatial navigation—whether it involves crawling through tunnels, maneuvering around obstacles, climbing over items, rocking on a horse, or pushing a toy cart. Such activities not only aid children in understanding their bodies’ movements in relation to their surroundings but also positively affect their motor skills [44,45]. Therefore, gross motor toys are indispensable in supporting the motor development of infants.

### 4.7. Risk and Protective Factors Associated with Children’s Cognitive Development: Length of Breastfeeding

The hierarchical regression analysis revealed significant findings concerning cognitive outcomes across all three models. Initially, in the first two models, factors such as the length of breastfeeding, mechanical ventilation, and teachers’ experience were identified as explanatory variables for cognitive variance. However, when controlling for all other factors, only the length of breastfeeding remained significant in the final model, explaining 24% of the variance in cognitive development. Children breastfed for longer durations (7 months) exhibited higher cognitive scores compared to those who were breastfed for shorter periods (five months or less).

Strong previous research showed that breast milk plays a crucial role in promoting cognitive development during infancy and early childhood, largely due to its unique and bioactive composition. It contains essential long-chain polyunsaturated fatty acids (such as DHA and ARA), which are fundamental for brain development, particularly in the formation of neuronal membranes and synaptic function. Breast milk is also rich in lactose, which supports brain energy metabolism, and contains hormones, growth factors, and immune-modulating agents that contribute to optimal neurodevelopment [46].

Additionally, research consistently highlights the protective role of breastfeeding in cognitive development, for example in problem solving and memory, with enduring effects across childhood [30,47,48,49]. Moreover, breastfeeding’s positive impact on cognitive development persists, even after adjusting for maternal educational level [47,50], consistent with the findings of the present study. A plausible explanation for the association between breastfeeding duration and enhanced cognitive scores lies in the increased frequency and duration of interactions between mothers and children. These interactions promote direct verbal vocalizations [47], thereby influencing the child’s responsiveness to stimuli.

### 4.8. Risk and Protective Factors Associated with Children’s Language Development: Teachers’ Knowledge and Children’s Enrollment in Private or Public ECCs

Regarding language outcome, the results from the hierarchical regression showed that all three models were significant. Initially, in the first two models, the teachers’ experience explained the variance. However, when controlling for all other factors, the ones that remained significant in the last model were teachers’ knowledge about children’s development and children attending public ECCs, explaining 31% of the variance in language development.

Teachers’ knowledge about child development plays a crucial role in shaping interactions and creating enriched environments encouraging language development [10,51]. Such knowledge enables teachers to interact more sensitively, understand children’s needs better, and support their development in ECC environments. Specifically, teachers with a deeper understanding of developmental milestones and principles are likely better equipped to distinguish between typical and atypical language skills, facilitating early identification and support for children who may require additional assistance or directing families towards further services.

The distinction between children attending public and private ECCs emerges as a significant factor influencing language scores, with children in public ECCs exhibiting higher language development compared to those in private ECCs. This discrepancy likely arises from various factors, including differences in teacher quality, ECC physical environments, SES, and breastfeeding duration. Children in public ECCs benefit from several protective factors, particularly longer breastfeeding periods at home, which have been associated with better language outcomes [48]. Additionally, they are exposed to high-quality teaching practices and favorable indoor physical spaces for play and exploration, factors that nurture development [12,14]. These factors collectively support language acquisition in children attending public ECCs.

It is important to acknowledge that families enrolling their children in public ECCs typically have lower monthly incomes and predominantly belong to low SES backgrounds. Many parents work in manual labor jobs with hourly wages, and in several cases, the mothers serve as the primary providers, necessitating longer hours of ECC attendance for their children. Despite these socioeconomic challenges, the present results suggest that public ECCs play a crucial role in extending family education and providing compensatory opportunities for language development.

## 5. Strengths, Limitations, and Recommendations for Further Research

Children attending daily ECCs are susceptible to several risk and protective factors, leading to different developmental outcomes, and this intricate process was investigated in the present study. Examining sociocultural variables (i.e., SES, family income, teachers’ and assistants’ knowledge, teachers’ daily practices) in the context of collective non-parental childrearing is also a strength in the present study. However, there is still a considerable gap of evidence regarding how teachers’ knowledge is related to child development in the first two years of life. Moreover, investigating how ECC opportunities for children’s development are organized to nurture child development is another strength of this study. Furthermore, this study enrolled children from low- and middle-income families in an LMIC; research for children from LMIC countries—most of the world—is still scarce. Children’s exposure to risks and the diversity of sociocultural factors lead to different trajectories of development in LMICs; these factors need to be examined, and specific strategies for ECCs must be implemented considering this cultural and economic diversity.

This study had several limitations. First, the study examined teachers’ practices and knowledge. However, it was limited regarding exploring other domains of teachers’ behaviors on which teacher knowledge may have an impact, such as demonstration of affection, empathy to the child’s needs, dyads of interaction, and use of disciplinary actions. Also, the mother/child behaviors that occur during breastfeeding that could affect child development were not examined; we only examined the duration of breastfeeding. Research must address this issue so that there can be a better understanding of potentially protective factors that affect cognitive, motor, and language development in the first years of life. Therefore, future research should also address teachers’ and mothers’ daily behaviors more in-depth; qualitative approaches may be exciting to these factors. Second, although it was not the focus of the present study, a longitudinal design addressing professional practice and knowledge and the impact on child development could provide insight into this complex, multifactorial process of childrearing in collective environments. Third, we did not control for the effect of families’ quality of care provided to the children when they returned home at the end of the day. It is essential to highlight that the children stayed eight hours in the ECC(s), with the time at home for the involvement of parents in child stimulation processes restricted to nights and weekends. However, responsive parents, even for a short time, can enhance child development; consequently, it was a limitation in the present study. Fourth, although our sample size was not small, it was insufficient to further subdivide the sample in the analysis (i.e., children with and without cognitive delays, language, and motor delays in public and private centers). While this was not the objective of our study, such analysis could provide additional insights into child development, and we recommend that future studies explore these comparisons.

## 6. Conclusions

The results highlight the need for guidelines and investments to improve the quality of children’s experiences in the ECCs, with different priorities for public and private centers. Unambiguously in the present study, it is necessary to improve teachers’ and assistants’ knowledge about children’s development (means scores around or below 50% correct answers), enhance their practices, and provide appropriate outdoor spaces for children in public ECCs and indoor spaces for children in private ECCs. These findings reinforce the need for surveillance and promotion of educational opportunities for children at ECCs, with critical attention to those more susceptible to risks or manifesting delays. This assumption is even more relevant to low-income families, as their children are more likely to have combined risk factors due to lower SES, fewer home opportunities for development, and mothers who are more driven to the workforce. Public policies should focus on strongly subsidizing public ECCs, monitoring children’s development, promoting teachers’ accreditation and training, developing guidelines to support children’s full developmental potential, and consolidating the right of every child in an LMIC to access high-quality childcare. However, private centers must also be monitored by the educational council and receive incentives to improve the quality of care. Additionally, public policies should continue to encourage and support breastfeeding for as long as possible, given its well-established benefits for children’s cognitive, motor, and socioemotional development, as well as its role in reducing health inequalities and supporting maternal–child bonding. By outlining the intricate relationship between ECC environments and child development, particularly in the context of socioeconomic disparities, this study provided evidence to inform policy and practice, ensuring that all children, irrespective of background, have access to nurturing and developmental opportunities in their early years.

## Figures and Tables

**Table 1:** Children’s and families’ characteristics, professionals’ experience, knowledge and daily practice, and opportunities for development: public and private ECC comparisons.

Children, Families, Professionals, and ECCs Factors		Early Childcare Centers M (SD) ^1^ or n (%) ^2^		Group Comparisons ^3^	
		Public (n = 46)	Private (n = 38)	*p*	Cohen’s *f*
Children					
Age (months) ^1^		12.2 (3.0)	11.8 (3.9)	0.511	0.007
Sex ^2^	Boys	22 (47.8%)	19 (50%)	0.600	--
	Girls	24 (52.2%)	19 (50%)		
Ethnicity ^2^	White	33 (71.7%)	26 (68.4%)	0.843	--
	Black	8 (17.4%)	6 (15.8%)		
	Brown	5 (10.9%)	6 (15.8%)		
Gestational age (weeks) ^1^		38.3 (1.7)	38.8 (1.3)	0.125	0.182 ^#^
Mode of delivery, vaginal birth		22 (47.8%)	19 (50%)	0.843	--
Cesarean section		24 (52.2%)	19 (50%)		
Preterm gestation		7 (15.2%)	5 (13.1)	0.127	--
Birth height (centimeters) ^1^		48.6 (2.4)	48.8 (1.7)	0.729	0.045
Birth weight (grams) ^1^		**3005.1 (544.6)**	**3287.0 (448.3)**	**0.017**	**0.291 ^##^**
APGAR 5^0^ min ^1^		8.7 (0.81)	8.7 (0.77)	0.709	0.045
Neonatal intensive care unit (days) ^1^		2.4 (7.6)	6.6 (18.3)	0.112	0.194 ^#^
Mechanical ventilation (days) ^1^		0.17 (0.82)	0.11 (0.65)	0.678	0.045
Breastfeeding (months) ^1^		**7.6 (4.7)**	**5.4 (2.5)**	**0.015**	**0.358 ** ^##^
**Families**					
Family monthly income BLR (Reals) ^1^		**4942.6 (3226.3)**	**6502.2 (3237.8)**	**0.031**	**0.251 ^##^**
SES classification ^2^	B1	8 (17.4%)	10 (26.3%)	**0.034**	--
	B2	17 (37.0%)	17 (44.7%)		
	C1	7 (15.2%)	8 (21.1%)		
	C2	14 (30.4%)	3 (7.9%)		
Mothers’ education ^2^	High school	39 (84.8)	28 (73.7)	0.277	---
	College	7 (15.2)	10 (26.3)		
**Teachers**					
Teachers’ age (years) ^1^		31.1 (2.5)	30.1 (3.1)	0.129	0.172 ^#^
Teachers’ experience (years) ^1^		6.9 (2.2)	6.2 (3.1)	0.214	0.140 ^#^
Teachers’ daily practice (DAIS) ^1^		**20.4 (3.0)**	**18.9 (2.8)**	**0.021**	**0.268 ^##^**
Teachers’ knowledge (KIDI) ^1^		68.8 (12.4)	70.6 (9.6)	0.093	0.191 ^#^
**Assistants**					
Assistants’ age (years) ^1^		28.8 (2.9)	28.2 (3.2)	0.293	0.115 ^#^
Assistants’ experience (years) ^1^		6.0 (2.0)	5.4 (2.7)	0.232	0.133 ^#^
Assistants’ knowledge (KIDI) ^1^		57.6 (10.7)	57.2 (14.8)	0.617	0.054
**ECC(s)’ opportunities for development (ADEMD) ^1^**					
Indoor space ^1^		**2.7 (1.2)**	**2.4 (1.0)**	**0.158**	**0.159 ^#^**
Outdoor space ^1^		**3.3 (1.2)**	**4.7 (1.4)**	**<0.001**	**0.910 ^###^**
Variety of stimulation ^1^		21.9 (1.7)	21.6 (1.2)	0.424	0.090
Fine motor toys ^1^		24.2 (4.6)	23.5 (3.2)	0.448	0.084
Gross motor toys ^1^		15.0 (3.0)	15.6 (2.1)	0.264	0.124 ^#^

Note: ^1^ Mean and standard deviations; ^2^ number and percentage; ^3^ group comparisons: one-way ANOVA and chi-square; significant results in bold; KIDI: Knowledge of Infant Development Inventory; DAIS: Daily Activities of Infant Scale; Cohen’s *f* effect size: *f* small = 0.10 to 0.24 ^#^, *f* moderate = 0.25 to 0.39 ^##^, *f* large ≥ 0.40 ^###^.

**Table 2:** Public and private ECC comparisons: children’s development.

Children’s Development	Early Childcare Centers M (SD)		*p* ^1^	Cohen’s *f*
	Public (n = 46)	Private (n = 38)		
**BSITD III Composite Score**				
Motor development	84.5 (15.3)	81.8 (13.8)	0.400	0.096
Cognitive development	**93.4 (12.4)**	**88.2 (10.5)**	**0.044**	**0.230 ^#^**
Language development	**84.5 (13.9)**	**79.0 (10.7)**	**0.050**	**0.211 ^#^**

Note: Grupo comparisons: ^1^ one-way ANOVA; significant results are in bold; Cohen’s *f* effect size: *f* small = 0.10 to 0.24 ^#^.

**Table 3:** Correlation matrix between all variables that remained in the model.

Variables	1	2	3	4	5	6	7	8	9	10	11	12	13	14	15	16	17	18
1 Age	-																	
2 Birth weight	−0.01	-																
3 Gestational age	−0.46 ***	0.87 ***	-															
4 Mech. Ventilation	0.12	−0.10	−0.12	-														
5 Breastfeeding	0.03	0.13	0.09	−0.00	-													
6 DAIS Teachers	0.34 **	−0.02	−0.10	0.02	0.15	-												
7 KIDI Teachers	−0.05	0.14	−0.15	0.12	0.10	0.15	-											
8 KIDI Assistants	−0.03	0.22	−0.08	0.12	0.24 *	0.22 *	0.68 ***	-										
9 Teachers’ experience	0.49 ***	0.08	0.18	−0.18	−0.14	0.08	−0.41 ***	−0.39 ***	-									
10 Mother Edu ^a (College)^	0.01	0.03	0.02	−0.09	−0.18	0.11	−0.16	−0.09	00	-								
11 Indoor space	0.31 **	−0.05	−0.25 *	0.08	−0.14	0.27 *	0.13	0.31 **	0.06	0.20	-							
12 Outdoor space	−0.11	0.09	−0.09	0.05	−0.23 *	−0.24 *	−0.03	0.06	−0.19	**0.** 30 **	0.05	-						
13 Fine motor toys	0.35 **	−0.06	−0.37 ***	0.27 *	0.15	0.19	0.05	0.03	−0.02	0.11	0.20	−0.20	-					
14 Gross motor toys	0.48 ***	0.03	−0.43 ***	0.28 **	0.11	−0.04	0.21	0.18	0.00	0.12	0.42 ***	0.36 ***	0.35 **	-				
15 Monthly income	−0.18	0.03	0.16	−0.00	−0.07	−0.13	−0.08	0.18	−0.04	0.07	−0.11	0.01	0.07	−0.19	-			
16 ECC(s) ^(public)^	−0.06	0.23 *	0.03	−0.05	−0.26 *	−0.26 *	0.21	0.08	−0.11	0.14	−0.15	0.63 ***	−0.26 *	0.21	0.22 *	-		
17 Motor	0.75 ***	0.13	−0.35 ***	0.13	0.19	0.29 **	0.08	0.07	0.32 **	0.07	0.25 *	−0.15	0.39 ***	0.48 ***	−0.06	−0.09	-	
18 Cognitive	0.40 ***	0.20	−0.13	0.07	0.33 **	0.26 *	0.15	0.14	0.14	−0.03	0.17	−0.25 *	0.37 ***	0.18	−0.03	−0.19	0.57 ***	-
19 Language	0.41 ***	0.10	−0.33 ***	0.163	0.25 *	0.30 **	0.31 ***	0.35 ***	−0.02	0.00	0.29 ***	−0.10	0.37 ***	0.39 ***	−0.14	−0.21	0.64 ***	0.46 ***

Note: Mech. Ventilation: mechanical ventilation; Mother Edu: mothers’ education; ECCs: Early Childcare Centers; * *p* < 0.050, ** *p* < 0.010, *** *p* < 0.001, ^a^ reference category.

**Table 4:** Hierarchical linear regression associations for motor, cognitive, and language outcomes.

Variables	Motor				Cognitive				Language			
Model 1	B	SE	*p*	β	B	SE	*p*	β	B	SE	*p*	β
Age	−12.9	7.6	0.095	−0.24	0.19	5.89	0.974	0.00	−8.28	6.48	0.206	−0.18
Birth weight	−0.00	0.00	0.743	−0.07	−0.00	0.00	0.612	−0.11	0.00	0.01	0.391	0.19
Gestational age	2.50	2.0	0.210	0.29	2.03	1.53	0.191	0.29	−0.57	1.69	0.738	−0.08
Mech. Ventilation	2.94	2.5	0.246	0.14	3.56	1.95	0.072	0.21	2.55	2.14	0.238	0.14
Breastfeeding	1.30	0.53	**0.017**	0.28	1.51	0.41	**<** **0.001**	0.39	1.56	0.46	**0.001**	0.38
**Model 1 FIT**	Adj R^2^ = 0.08; *f*^2^ = 0.09 ^#^; AIC = 611.4; BIC = 627.7				Adj R^2^ = 0.19; *f* ^2^ = 0.19 ^#^; AIC = 572.6; BIC = 588.9				Adj R^2^ = 0.14; *f*^2^ = 0.16 ^##^; AIC = 587.0; BIC = 603.3			
**Model 2**	**B**	**SE**	***p***	**β**	**B**	**SE**	***p***	**β**	**B**	**SE**	***p***	**β**
Age	−11.0	6.3	0.08	−0.20	0.91	5.71	0.874	0.02	−7.88	6.07	0.198	−0.17
Birth weight	−0.00	0.00	0.590	−0.10	−0.00	0.00	0.786	−0.06	0.00	0.01	0.600	0.11
Gestational age	2.71	1.64	0.106	0.31	1.87	1.50	0.216	0.26	−0.24	1.59	0.879	−0.03
Mech. Ventilation	4.80	2.09	**0.025**	0.23	4.35	1.90	**0.025**	0.25	2.81	2.02	0.169	0.15
Breastfeeding	1.25	0.46	**0.008**	0.27	1.43	0.42	**0.001**	0.38	1.23	0.44	**0.008**	0.30
DAIS Teachers	0.50	0.45	0.275	0.11	0.76	0.41	0.073	0.20	0.56	0.44	0.212	0.14
KIDI Teachers	0.11	0.13	0.407	0.09	0.14	0.13	0.279	0.14	0.22	0.13	0.102	0.21
KIDI Assistants	0.04	0.16	0.762	0.03	−0.16	0.15	0.288	−0.15	0.21	0.16	0.182	0.19
Teachers’ Experience	2.92	0.53	**<0.001**	0.58	0.97	0.48	**0.049**	0.23	1.30	0.51	**0.014**	0.29
Mother Edu. ^a (College)^	2.84	3.23	0.382	0.21	−0.08	2.93	0.978	−0.01	2.40	3.12	0.444	0.20
**Model 2 FIT**	Adj R^2^ = 0.38; ΔR^2^ (models 1 and 2) = 0.33 ***; *f*^2^ = 0.48 ^##^; AIC = 585.2; BIC = 613.2				Adj R^2^ = 0.25; ΔR^2^ (models 1 and 2) = 0.11; *f*^2^ = 0.08 ^#^; AIC = 570.4; BIC = 598.4				Adj R^2^ = 0.26; ΔR^2^ (models 1 and 2) = 0.17 **; *f*^2^ = 0.16 ^##^; AIC = 579.5; BIC = 607.5			
**Model 3**	**B**	**SE**	***p***	**β**	**B**	**SE**	***p***	**β**	**B**	**SE**	***p***	**β**
Age	−9.07	6.46	0.165	−0.17	0.46	6.05	0.940	0.01	−2.89	6.18	0.642	−0.06
Birth weight	−0.00	0.01	0.775	−0.06	−0.00	0.01	0.946	−0.02	0.01	0.01	0.158	0.34
Gestational age	2.60	1.85	0.166	0.30	1.81	1.74	0.300	0.26	−1.62	1.77	0.366	−0.21
Mech. Ventilation	3.68	2.29	0.113	0.18	4.21	2.14	0.054	0.25	0.85	2.19	0.699	0.05
Breastfeeding	0.76	0.55	0.176	0.16	1.27	0.52	**0.017**	0.33	0.38	0.53	0.471	0.09
DAIS Teachers	1.03	0.50	**0.046**	0.23	0.55	0.47	0.248	0.15	0.68	0.48	0.164	0.17
KIDI Teachers	0.08	0.15	0.589	0.07	0.15	0.14	0.314	0.15	0.33	0.15	**0.030**	0.31
KIDI Assistants	−0.01	0.20	0.980	−0.00	−0.12	0.19	0.513	−0.12	0.16	0.19	0.409	0.14
Teachers’ Experience	2.40	0.60	**<0.001**	0.47	0.87	0.56	0.128	0.21	1.10	0.58	0.062	0.25
Mother Edu ^a (College)^	2.91	3.38	0.393	0.22	1.08	3.16	0.734	0.10	1.65	3.23	0.612	0.14
ECC indoor space	−2.46	1.53	0.114	−0.21	−0.32	1.44	0.826	−0.03	−0.56	1.47	0.706	−0.05
ECC outdoor space	−2.73	1.71	0.116	−0.25	−1.41	1.60	0.381	−0.16	0.77	1.64	0.639	0.08
Fine motor toys	−0.31	0.40	0.442	−0.09	0.30	0.37	0.423	0.10	−0.14	0.38	0.720	−0.04
Gross motor toys	2.40	0.93	**0.012**	0.41	−0.06	0.87	0.946	−0.01	1.36	0.89	0.130	0.27
Monthly income	0.00	0.00	0.140	0.16	0.00	0.00	0.787	0.03	−0.00	0.00	0.706	−0.04
ECC(s) ^a (public)^	0.51	3.66	0.890	0.04	1.91	3.43	0.579	0.17	8.87	3.50	**0.014**	0.75
**Model 3 FIT**	Adj R^2^ = 0.41; ΔR^2^ (models 2 and 3) = 0.07; *f*^2^ = 0.05 ^#^; AIC = 586.0; BIC = 627.9				Adj R^2^ = 0.24; ΔR^2^ (models 2 and 3) = 0.05; *f*^2^ = −0.01 ^#^; AIC = 576.0; BIC = 618.0				Adj R^2^ = 0.31; ΔR^2^ (models 2 and 3) = 0.10; *f*^2^ = 0.07 ^#^; AIC = 579.2; BIC = 621.2			

Note: Mech. Ventilation: mechanical ventilation; Mother Edu: mothers’ education; ECC(s): Early Childcare Center(s); B: unstandardized estimative; SE: standard error; β: standardized; *p:* probability value; Adj R^2^: adjusted R^2^; ^a^ reference category; *f*^2^ effect size for the hierarchical multiple regression: ^#^ small effect size, ^##^ moderate effect size. ** *p* < 0.010, *** *p* < 0.001.

## Data Availability

The raw data supporting the conclusions of this article will be made available by the authors upon request.
